# The effect of chiropractic care on infantile colic: results from a single-blind randomised controlled trial

**DOI:** 10.1186/s12998-021-00371-8

**Published:** 2021-04-19

**Authors:** Lise Vilstrup Holm, Dorte Ejg Jarbøl, Henrik Wulff Christensen, Jens Søndergaard, Lise Hestbæk

**Affiliations:** 1 The Chiropractic Knowledge Hub, University of Southern Denmark, Campusvej 55, DK-5230 Odense M, Denmark; 2 Research Unit of General Practice, Department of Public Health, University of Southern Denmark, J.B. Winsløws vej 9A, DK-5000 Odense C, Denmark; 3 Department of Sports Science and Clinical Biomechanics, University of Southern Denmark, Campusvej 55, 5230 Odense C, Denmark

**Keywords:** Infantile colic, Chiropractic, Randomized controlled trial, Manipulative treatment, Excessive crying

## Abstract

**Background:**

Chiropractic care is commonly used to treat infantile colic. However large trials with parental blinding are missing. Therefore, the purpose of this study is to evaluate the effect of chiropractic care on infantile colic.

**Method:**

This is a multicenter, single-blind randomized controlled trial conducted in four Danish chiropractic clinics, 2015–2019. Information was distributed in the maternity wards and by maternal and child health nurses. Children aged 2–14 weeks with unexplained excessive crying were recruited through home visits and randomized (1:1) to either chiropractic care or control group. Both groups attended the chiropractic clinic twice a week for 2 weeks. The intervention group received chiropractic care, while the control group was not treated. The parents were not present in the treatment room and unaware of their child’s allocation.

The primary outcome was change in daily hours of crying before and after treatment. Secondary outcomes were changes in hours of sleep, hours being awake and content, gastrointestinal symptoms, colic status and satisfaction. All outcomes were based on parental diaries and a final questionnaire.

**Results:**

Of 200 recruited children, 185 completed the trial (treatment group *n* = 96; control group *n* = 89). Duration of crying in the treatment group was reduced by 1.5 h compared with 1 h in the control group (mean difference − 0.6, 95% CI − 1.1 to − 0.1; *P* = 0.026), but when adjusted for baseline hours of crying, age and chiropractic clinic, the difference was not significant (*P* = 0.066). The proportion obtaining a clinically important reduction of 1 h of crying was 63% in the treatment group and 47% in the control group (*p* = 0.037), and NNT was 6.5. We found no effect on any of the secondary outcomes.

**Conclusion:**

Excessive crying was reduced by half an hour in favor of the group receiving chiropractic care compared with the control group, but not at a statistically significant level after adjustments. From a clinical perspective, the mean difference between the groups was small, but there were large individual differences, which emphasizes the need to investigate if subgroups of children, e.g. those with musculoskeletal problems, benefit more than others from chiropractic care.

**Trial registration:**

Clinical Trials NCT02595515, registered 2 November 2015

## Background

Infantile colic is a common condition that occurs in up to 25% of infants and is characterized by excessive crying and fussing in infants who thrive and develop normally in all other aspects [1]. Wessel was the first to define the condition with the ‘rule of three’, that is, where the infant was crying at least 3 h a day, for at least 3 days a week in the previous 3 weeks [2]. Modifications of Wessels have included slightly different timeframes for the condition over time, but the cornerstone of excessive crying and fussing are unaltered in the new Rome IV criteria, with the addition that the babies are less than 5 months of age when symptoms start and stop [3].

Although infantile colic is considered a self-limiting condition, it is often very stressful which leads carers to seek interventions. Much research has been conducted to establish its underlying cause, but the pathophysiology remains unclear. A condition in the gastrointestinal system has been suggested, probably reflecting the typical behaviour the child exhibits during a crying episode, characterised by drawing up the legs to the stomach with a face that appears in pain [4]. Hypotheses of causes of gastrointestinal origin include an allergy to cows’ milk, immaturity of the intestines, and a different intestinal microflora [4–7], while hypotheses concerning non-gastrointestinal causes include prenatal factors, birth factors, biomechanical strains and disturbances in the parent-child relationship, among others [8–12].

Many interventions have been investigated over time, but it has been difficult to point out one specific treatment modality [5, 12–14]. Hence, no standardized treatment exists for infantile colic, possibly reflecting the unclear pathophysiology. One of the treatments parents seek is chiropractic care, and children worldwide are increasingly diagnosed and treated in chiropractic clinics [15]. A Danish study showed that infantile colic was the most frequent inquiry for infants treated in chiropractic clinics, and the use of chiropractic care for infants in Denmark has almost tripled in the last decade [16, 17]. In contrast to this, the effect of chiropractic care on infantile colic has only been sparsely investigated. A Cochrane review regarding manipulative therapies for infantile colic identified six randomized trials involving a total of 325 infants [18]. The most common primary outcome was change in daily hours of crying. Of the six studies, five showed a positive effect on daily hours of crying, and one found no difference when compared with the natural history of infantile colic. However, in general the studies were small and, in most cases, lacked parental blinding, thereby increasing the risk of performance bias. When only considering the studies with parental blinding, there was a smaller and non-significant reduction in crying time in the manipulative group compared with the control group. In 2018, Carnes et al. repeated the meta-analysis on available RCTs evaluating the effect of manual therapy on crying time for unsettled, distressed and excessively crying infants [19]. They found evidence of moderate strength for a small positive effect on crying time, with the same reservations as the Cochrane Review, and with the conclusion that future research should comprise well-powered studies with parental blinding. We carried out a randomized controlled trial with parental blinding on a larger scale to evaluate the effect of chiropractic care on infantile colic. The protocol of the study has been published elsewhere [20].

## Methods

### Trial design

This was a multicenter, single-blind, randomized [1:1] controlled trial.

### Participants, setting and procedures

Children were recruited at the age of 2 to 14 weeks with symptoms of infantile colic defined as episodes of excessive crying and fussing that lasted at least 3 h a day, for at least 3 days a week in the previous 2 weeks. The children were otherwise healthy and thriving with appropriate weight gain. The children were ineligible if they had received chiropractic treatment before. Concomitant treatment for colic (e.g. reflexology) was not permitted during the project period.

The study was carried out from November 2015 to July 2019 on the Island of Funen in Denmark (Approximately 500,000 inhabitants and 4600 births per year). Parents of newborns were informed about the existence of the project through maternal and child health nurses, the local maternity wards, the participating chiropractic clinics and through the media. The parents themselves contacted the primary investigator (PI) by telephone, who screened the child and arranged a home visit if criteria seemed to be fulfilled. The screening included questions to assess if the child cried excessively but besides that seemed healthy and thriving. If the child, based on the answers, were suspected not to thrive, e.g. if the weight gain was too small, the parents were advised to contact their maternal and child health nurse and/or their general practitioner.

On the first visit, the PI assessed the child more thoroughly for eligibility, including a basic medical examination. Parents were informed of the study and gave written consent to participate. The PI completed an interview-based questionnaire with the purpose being to collect baseline information about the child and family [20]. Parents were instructed to keep a 24-h diary of their child’s behavior throughout the two-week project period, a method that has been validated and proved reliable [21]. Every page in the diary represented 24 h, divided into time intervals of 15 min [20]. These were filled out with a variety of symbols representing different aspects of the child’s behavior, including the amount of inconsolable crying (crying without an obvious reason that could not be comforted by the parents), the time the child needed to be held and rocked to limit crying (crying without any obvious reason that was only partly and briefly limited if the child was constantly held and rocked), the time the child was awake and content, the time spent sleeping, feeding patterns and bowel movements. Consolable crying with an obvious reason where the child was easily comforted e.g. hunger, or a soiled diaper was recorded as ‘awake and content’. To limit recall bias and make the recording of information as precise as possible, the parents were advised to fill in the diary several times a day, e.g. every time the baby was fed. Baseline recording of the crying pattern was done for at least 3 days whereupon a second visit from the PI was scheduled. Furthermore, the first chiropractic visit was pre-booked, but could be cancelled again if the child cried less than anticipated in the baseline observation period, thereby not fulfilling the inclusion criterion, or if the parents withdrew their consent.

For practical purposes, the second visit between the PI and the parents was often held in an office made available in the chiropractic clinic immediately before the scheduled first chiropractic visit. Here, any difficulties filling out the diary were identified, and the parent’s assessment of the crying pattern was evaluated in a second interview-based questionnaire [20]. Parents were asked to contact the PI and the treating chiropractor at any time during the project period if they suspected any adverse effects.

The eligible children were then randomized in a balanced 1:1 ratio to treatment or control group using computer-generated allocations with blocks of 4 to 6. A stratification was made according to the participant’s age at enrollment (2–6 weeks; 7–10 weeks; 11–14 weeks) and the treating chiropractor. Group assignment was noted in opaque envelopes and sent to the project clinics. The procedure was handled by the data manager at the Nordic Institute for Chiropractic and Clinical Biomechanics. Parents were blinded to the child’s allocation during the project period. Blinding the treating chiropractor was obviously not possible. For the research group including the statistician, the randomization code was first revealed after the analyses were completed. The randomization method was described in detail in the study protocol [20].

### Intervention

Four chiropractic clinics, involving one to three chiropractors from each clinic, participated in the study. All the chiropractors had a special interest and experience in pediatric practice and had been working for between 10 and 30 years in the field.

All children attended the chiropractic clinic two times a week for 2 weeks. The chiropractor obtained responses to selected case history questions of possible musculoskeletal problems from all participants through an interview-based questionnaire [20]. To ensure blinding of the parents, they were then asked to leave the room. For both groups, the chiropractor then undressed the child and observed if there were any visible asymmetries that could indicate a dysfunction in the musculoskeletal system. Children in the control group received no active treatment but were entertained for 5 min to mimic the time of treatment and were then dressed again. For children in the intervention group a full examination, including movement palpation of the joints was carried out, and the manual therapy applied was individualized according to any biomechanical dysfunction found. Thus, the chiropractors were informed that manual therapy could include manipulation or mobilization of the spine and/or the extremities as they found indicated by the child’s potential biomechanical dysfunctions, including movement restriction, tenderness or an obvious asymmetry in the muscles or joints. The treatment technique for restricted joint movements in this age group is, in general, very light short-term pressure with fingertips and gentle massage in case of hypertonic muscles. Specific advice directed towards any biomechanical dysfunction, and explanation of exercises that supported the effect of the manual therapy should be provided to the parents in the intervention group, while parents in the control group could only receive pragmatic advice such as initiating a cycling movement of the child’s legs.

After the fourth visit, the parents continued their record-keeping in the diary for 1 to 4 days and filled out a final questionnaire [20]. The parents then attended the clinic again to hand in the documents and receive information about their child’s group allocation. All treatments were free of charge to the families (trial-funded), and parents of infants from the control group were immediately offered four treatments free of charge following the study period.

### Outcome measures

Hours of crying were defined as inconsolable crying and the amount of time the child needed to be held and rocked to limit crying based on the record in the parental diaries. Daily hours of crying before treatment were defined as the average number of hours of crying recorded during the baseline observation period over at least 3 days. Daily hours of crying after treatment were defined as the average number of hours of crying recorded over 1 to 4 days after the fourth visit to the chiropractic clinic.

The primary outcome measure was defined as the change in daily hours of crying before and after treatment.

In addition, reduction of at least 1 h of crying (yes/no) was defined a priori as a clinically meaningful reduction.

Hours of sleep, and hours when awake and content, were based on the record in the parental diaries in the same way as daily hours of crying. Secondary continuous outcomes were changes in hours of sleep and hours when awake and content. Secondary categorical outcomes were obtained from parental final questionnaires and included changes in bowel movements (no changes/more often/more rarely; no changes/easier/more difficult), burps (no changes/easier/more difficult), hiccups (no changes/more often/less often, regurgitation (no changes/more/less), satisfaction with participation (yes/no), and status of the colic (dichotomized into stopped/decreased or unchanged/increased).

Parents were advised to immediately contact the chiropractor and/or the PI if they suspected any side effects or adverse events during the project period.

### Sample size

A sample size calculation was conducted when the data collection was completed for 23 children in total. This was based on daily hours of crying before and after the treatment period, and details are given in the study protocol [20]. A sample size of 100 children in each group was indicated to detect a difference of 1 h of crying with a power of 80% and a significance level of 5%.

### Statistical analyses

All analyses were done according to intention-to-treat principles. No data were imputed.

First, we compared the primary outcome between randomization groups using a two-sample t-test on the individual differences. Second, we analyzed group effect on the primary outcome in a multiple linear regression, adjusting for primary outcome at baseline (model 1) and additionally for age (continuous, in weeks) and clinic (categorical) in model 2. Continuous data on secondary outcomes were analyzed similarly.

For the binary (yes/no) reduction of daily hours of crying for more than 1 h, we compared the proportions improved between the groups, including a test for statistical significance by means of a Chi square test, and estimated the difference between proportions and numbers needed to treat (NNT).

Categorical data on secondary outcomes from the parent-reported questionnaire were analyzed by Chi-squared tests.

The success of blinding was reported as the proportion believing to belong the active treatment group for both groups, and the association with outcome was tested with linear regression analysis.

Throughout the study, a *p*-value below .05 was considered statistically significant. Analyses were performed using Stata Release 16 (StataCorp, College Station, TX, USA).

### Amendments to study protocol

The age criterion for inclusion was extended from 2 to 10 weeks to 2–14 weeks within the first half year of the trial since children older than 10 weeks were referred. Furthermore, an additional chiropractic clinic was included to accommodate participants geographically. In order to take these factors into account the randomization scheme was optimized, so randomization thereafter was stratified according to the child’s age and treating chiropractor. The changes were reported as amendments to the study protocol in April 2016 and approved by the Regional Committee on Health Research Ethics.

## Results

### Study population

**Fig. 1:**
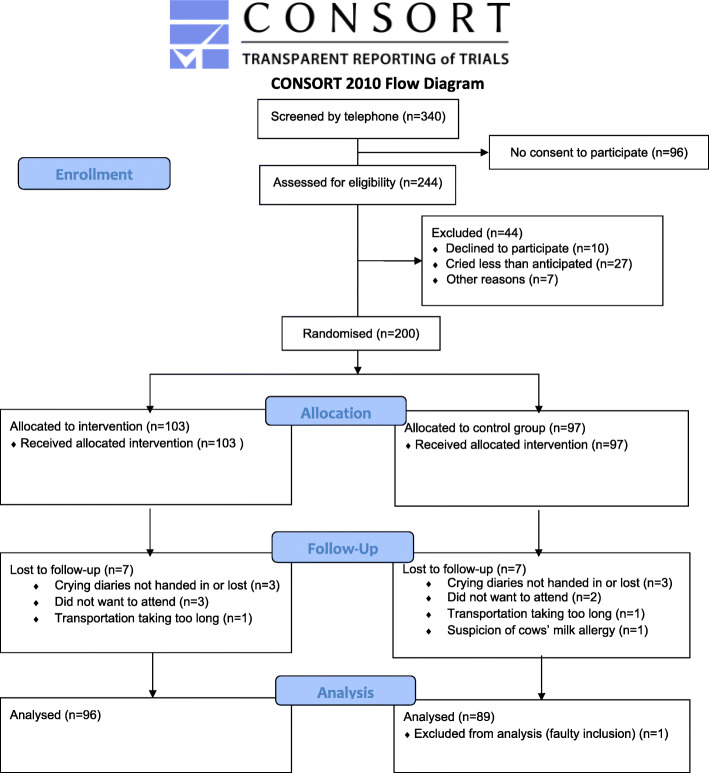
Consort flow chart

The inclusion period lasted from November 5, 2015 to July 22, 2019. Of the 340 children screened by telephone, 244 received a first visit (Fig. 1). During the baseline observation period, 44 children were excluded, most often because the child cried less than anticipated before entering the trial. Of the 200 children randomized to either treatment or control group, 185 completed the trial with a full dataset and were included in the analyses. One child was excluded from the analyses due to a faulty inclusion (5 days too old according to the inclusion criterion). Missing data and dropouts were equally distributed between the treatment and the control groups. In total there were missing data on six children. Eight children in total dropped out after randomization. One of the withdrawals was based on the parents’ suspicion of an adverse event to treatment (due to increased crying the day after the first chiropractic visit), however it was revealed that this child was allocated to the control group. The remaining reasons for withdrawals included one case with suspicion of an allergy to cows’ milk protein, which needed to be further investigated, two families had difficulties with transportation to the clinic, and four families could not accept the uncertainty of group allocation.

### Baseline characteristics

There were no pronounced differences in baseline characteristics between the two groups, except for sex, with a greater proportion of girls in the treatment group. The educational level of the mothers showed slight differences between the groups (Table 1).

**Table 1:** Baseline characteristics of infants and their families. Results presented as absolute figures (percentages of total) or mean values (SD). Missing values comprised maximum 1% and were collapsed with no category

	All *N* = 185 (100 %)	Treatment group *N* = 96	Control group *N* = 89
Male	90 (48.6)	40 (41.7)	50 (56.2)
Female	95 (51.4)	56 (58.3)	39 (43.8)
Birth Weight (g)	3438 (583)	3452 (630)	3422 (529)
Birth length (cm)	51 (2.4)	51 (2.6)	51 (2.2)
Gestational age at birth (weeks)	39.5 (1.7)	39.5 (1.7)	39.5 (1.7)
Weight at study (g)	4742 (838)	4787 (874)	4694 (798)
Age at onset of colic (days)	13.1 (10.7)	13.1 (11.5)	13.2 (9.7)
Age at inclusion (weeks)	6.7 (2.8)	6.8 (2.9)	6.6 (2.6)
Age categories at inclusion (weeks)			
2-6	91 (49.2)	48 (50.0)	43 (48.3)
7-10	77 (41.6)	37 (38.5)	40 (44.9)
11-14	17 (9.2)	11 (11.5)	6 (6.7)
Feeding mode			
Breast-fed	104 (56.2)	52 (54.2)	52 (58.4)
Bottle-fed	51 (27.6)	28 (29.2)	23 (25.8)
Breast and bottle-fed	30 (16.2)	16 (16.7)	14 (15.7)
Birth			
Normal vaginal delivery	132 (71.4)	64 (66.7)	68 (76.4)
Vacuum-assisted delivery	8 (4.3)	5 (5.2)	3 (3.4)
Caesarean, planned	21 (11.4)	15 (15.6)	7 (7.9)
Caesarean, acute	24 (13.0)	13 (13.5)	11(12.4)
Siblings (yes)	105 (56.8)	56 (58.3)	49 (55.1)
Siblings with colic (yes)	35 (18.9)	16 (16.7)	19 (21.3)
Health of mother			
Physical or mental illness (yes)	48 (25.9)	26 (27.1)	22 (24.7)
Severe incident^a^ in the family during pregnancy (yes)	50 (27.0)	31 (32.3)	19 (21.3)
Severe incident^a^ in the family after birth (yes)	32 (17.3)	16 (16.7)	16 (18.0)
Educational level, mother			
Primary school	11 (5.9)	6 (6.3)	5 (5.6)
Upper secondary school	7 (3.8)	4 (4.2)	3 (3.4)
Skilled	21 (11.4)	18 (18.3)	3 (3.4)
Short academic education (2-2.5 years)	26 (14.1)	14 (14.6)	12 (13.5)
Medium academic education (3.5-4 years)	62 (33.5)	26 (27.1)	36 (40.4)
Long academic education (>5 years)	34 (18.4)	19 (19.8)	15 (16.9)
Undergoing education	24 (13.1)	9 (9.4)	15 (16.9)
Cohabitation status			
Cohabiting/married	172 (93.0)	89 (92.7)	83 (93.3)
Living alone	13 (7.0)	7 (7.3)	6 (6.7)

^**a**^death in family/among close friends, serious illness, unemployment, bankruptcy or similar

### Primary outcome, NNT and secondary outcomes

**Fig. 2:**
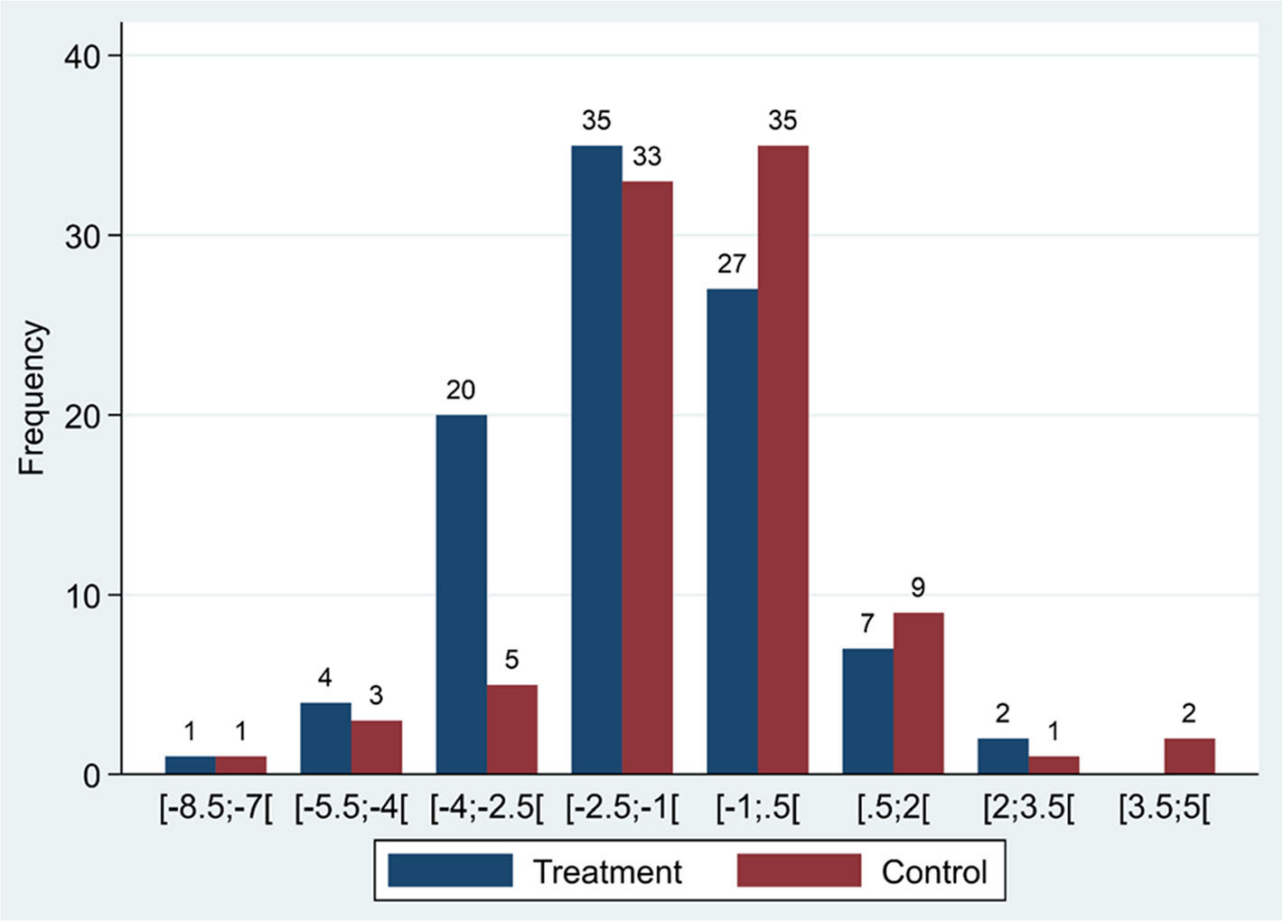
Post-pre differences in hours of crying are divided into groups, and number of infants belonging to each group are displayed by treatment group. X-axis: Hours of crying. Square brackets including interval of change in hours of crying for separate columns; numbers are included when bracket is facing towards the number and not included when turning away from the number

In both the treatment group and the control group, there was a reduction in hours of crying during the study period. In the treatment group, the mean reduction in duration of crying was 1.5 h compared with 1 h in the control group. The prespecified crude analysis showed that the mean difference between the groups was statistically significant (MD − 0.6, 95% CI .1.1 to − 0.6; *P* = 0.026) (Table 2). However, the difference did not remain statistically significant when adjusted for baseline hours of crying (*P* = 0.053) as well as age and chiropractic clinic (*P* = 0.066). The range of change in hours of crying varied from − 8.5 to + 3.5 and is illustrated by group in Fig. 2.

**Table 2:** Results for the primary outcome

	Treatment group *N* = 96	Control group *N* = 89	Difference in mean (95% CI)	*p*-value	*p*-value*	*p*-value**
Mean total hours of crying (95% CI)						
Before treatment	5.7 (5.2–6.2)	5.3 (4.9–5.8)	-0.6 (0.1–1.1)	0.026	0.054	0.066
After treatment	4.2 (3.8–4.7)	4.4 (3.8–4.9)				
Pre-post difference	−1.5 (− 1.9 to − 1.2)	−1.0 (− 1.3 to − 0.6)				

*N* Number of children; *CI* Confidence Interval; *: adjusted for baseline hours of crying; **: adjusted for baseline hours of crying, age and chiropractic clinic

Improvement in 1 h or more crying time by group shows that this was achieved by 63% and 47% in the treatment group and the control group, respectively (*P* = 0.037) (Table 3). NNT was estimated to be 6.5, indicating that 6.5 children on average needed to be treated to gain one additional improvement of 1 h in crying time.

**Table 3:** Numbers needed to treat based on improvement of at least 1 h of crying

	Improvement of at least one hour of crying		Total	NNT (CI)
	No	Yes		
Treatment group	36 (37.5)	60 (62.5)	96 (100.0)	6.5 (3.5–101.9)
Control group	47 (52.8)	42 (47.2)	89 (100.0)	
Total	83 (44.9)	102 (55.1)	185 (100.0)	

Risk of improvement for control (p0): 0.472. Risk of improvement for intervention (p1): 0.625. Risk difference (p1 - p0): 0.153. Newcombe Method 10 95% CI: 0.010–0.288

Regarding secondary outcomes, hours of sleep as well as hours when awake and content both showed a non-significant improvement in favor of the treatment group (Table 4), whereas there was no detectable difference in colic status, satisfaction with participation or any of the changes in the different gastrointestinal symptoms. Overall, more than 90% were satisfied with participation in the trial.

**Table 4:** Results of the two continuous secondary outcomes

	Treatment group *N* = 96	Control group *N* = 89	Difference in mean (95% CI)	*p*-value	*p*-value*	*p*-value**
Mean hours of sleep (95% CI)						
Before treatment	11.6 (11.1–12.1)	11.9 (11.4–12.3)	−0.2 (− 0–2; 0.7)	0.272	0.461	0.464
After treatment	12.4 (12.1–12.8)	12.5 (12.1–12.9)				
Pre-post difference*	0.9 (0.6–1.2)	0.6 (0.3–0.9)				
Mean hours when awake and content (95% CI)						
Before treatment	3.2 (2.8–3.5)	3.3 (3.0–3.7)	0.3 (−0.0; 0.6)	0.081	0.088	0.105
After treatment	4.0 (3.7–4.4)	3.9 (3.5–4.3)				
Pre-post difference	0.9 (0.6–1.1)	0.6 (0.4–0.8)				

*N* Number of children; *CI* Confidence Interval; *: adjusted for baseline hours of crying; **: adjusted for baseline hours of crying, age and chiropractic clinic

### Blinding

The blinding appears to be successful as 59.6% guessed the correct group. Data are included in Table 5. The belief of group-belonging appears to be related to outcome, as the group who believed themselves to be in the treated group, reported a mean reduction of crying of 1.54 h, whereas the corresponding figure for the other group was 0.98 (*p* = 0.03).

**Table 5:** Parent-reported categorical secondary outcomes obtained from final questionnaires

	Total	Treatment group	Control group	*p*-value
General perceived effect (*N* = 184)				0.850
Stopped or improved	120 (62.5)	62 (64.5)	58 (65.9)	
Unchanged or Worse	64 (34.8)	34 (35.4)	30 (34.1)	
Believe child was treated (*N* = 173)				0.342
Yes	97 (56.1)	53 (59.6)	44 (52.2)	
No	76 (43.9)	36 (40.4)	40 (47.6)	
Satisfied with participation (*N* = 179)				0.473
Yes	152 (92.7)	78 (91.8)	74 (93.7)	
No	12 (7.3)	7 (8.2)	5 (6.3)	
Change in frequency of bowel movements (*N* = 182)				0.476
No	90 (52.6)	48 (54.5)	42 (50.6)	
More frequent	42 (24.6)	23 (26.1)	19 (22.9)	
Rarer	39 (22.8)	17 (19.3)	22 (26.5)	
Change in trouble with bowel movements (*N* = 179)				0.247
No	94 (56.0)	50 (58.1)	44 (53.7)	
Easier	51 (30.4)	28 (32.6)	23 (28.0)	
More difficult	23 (13.7)	8 (9.3)	15 (28.0)	
Changes in burps (*N* = 181)				0.797
No	114 (68.3)	58 (67.4)	56 (69.1)	
Easier	45 (26.9)	23 (26.7)	22 (27.2)	
More difficult	8 (4.8)	5 (5.8)	3 (3.7)	
Changes in regurgitation (*N* = 181)				0.367
No	91 (54.5)	50 (58.1)	41 (50.6)	
More often	46 (27.5)	24 (27.9)	22 (27.2)	
Less often	30 (18.0)	12 (14.0)	18 (22.2)	

Results presented in absolute figures and (percentages of total) and p-value for difference between groups. *N* Number of children; missing values excluded from analyses

### Adverse events

Only one suspicion of an adverse event was reported, but this child was in the control group.

## Discussion

### Principal findings

The results of this randomized, controlled trial suggested an overall small positive effect of chiropractic care on infantile colic, but the clinical significance is debatable. We found a reduction in crying time of half an hour in favor of the treatment group, which was not statistically significant. However, a larger proportion of participants in the treatment group achieved a predefined clinically relevant reduction in crying time of 1 h at a statistically significant level; in short, more children improved in the treatment group, but the difference was small. The NNT to achieve a one-hour reduction in crying was 6.5. In the interpretation of the clinical relevance of these results, it must be taken into account that the control group was not untreated but received both attention and sensible general advice. Furthermore, there were no adverse effects registered and there is no other evidence-based treatment available for this group as a whole.

### Comparison with other studies

A meta-analysis of RCTs showed a positive effect on crying time of just over 1 h [18] which is somewhat larger than the half hour in the current study. The larger effect size may have been caused by a placebo effect in the treatment group, since parental blinding was not present in most of the included studies. When only including the two studies with parental blinding in the analysis, the results were similar to ours [18]. So, in that context, our result of an overall small positive effect of chiropractic treatment seems plausible. We found no significant differences between the groups in all our secondary outcomes, which is in line with other studies [19].

There is no clear consensus about the size of a clinically meaningful reduction in duration of crying [18, 19], but a priori, we decided on a mean difference of a one-hour reduction [20], and this was achieved by a larger proportion in the treatment group than in the control group (63% versus 47%). Only one other study set a priori a goal for a clinically meaningful reduction in crying time, defined as below 2 h per day and a 30% reduction or more, and calculated a NNT [22]. They found a significantly greater proportion of infants receiving chiropractic care showed a reduction in crying time (OR 6.33; 95 CI 1.54–26.00 and 44% versus 18% in the blinded treated and non-treated groups, respectively resulting in an NNT of 3. The authors themselves state that although the results were statistically significant, the wide CIs reflect the small sample sizes and variability in data, which further implies the difficulty in estimating the precise treatment effect in the target population [22]. The Cochrane review calculated the same numbers for included RCTs and found significant reductions in crying time in favor of the treatment group in all studies except one blinded study that had comparable reductions in the control group (39% versus 42%) [18]. So, again, lack of parental blinding in most studies may explain some of the diversity, but since differences between blinded studies also occurred, other reasons such selection bias and different intervention or sham techniques between the studies cannot be ruled out.

### Strengths and limitations of the study

To our knowledge, this is the largest single-blind randomized controlled trial within this area to date.

Blinding of chiropractors was obviously not possible due to the nature of the study, but the chiropractors did not assess the outcomes, which were all parent-reported, and the statistician and the research group were blinded throughout all analyses. There was no sign of parents breaking the randomization code, since the proportions guessing correct and incorrect groups were almost equal. Completely equal proportions are not likely to be obtained whenever a treatment effect is present, since the belief about randomization group is highly related to the outcome [23]. The study therefore meets the most pronounced limitations addressed in reviews of previous studies on this topic, which were small and lacked parental blinding [18, 19, 24].

Another strength of the study was that the manual treatment was individualized with attention to any specific biomechanical dysfunction the child might have, rather than being a standardized treatment given to all.

During the pilot phase, we experienced that the project period should be no longer than 2 weeks for the parents to accept the conditions of the study. So even though the chiropractors may have believed a longer follow up was indicated in some cases, this was not feasible under the circumstances and is a possible limitation of the study.

For practical reasons, the study was geographically limited to the island of Funen. The type of education among the mothers varied slightly with more skilled workers in the treatment group and more mothers with a mid-level academic education in the control group, however the average length of those educations was three to 4 years. Overall, the educational level did not vary substantially from the background population [25]. There was a greater proportion of girls in the treatment group, however sex is not known to be related to infantile colic [26].

### Adverse events

The two latest reviews on this subject pointed out the lack of safety reporting in prior trials [18, 19]. In our study, parents were advised to immediately contact the chiropractor and/or the PI if they suspected any side effects or adverse events during the project period. We only experienced one suspicion of an adverse event, but this child was in the control group where active treatment was not provided. In Denmark, it is obligatory to report adverse events of chiropractic treatments, and no serious or lasting side effects have ever been reported in infants following the types of treatment used in this trial [15]. Furthermore, no compensation claims have ever been made for this age group in Denmark [27].

### Implications/clinical interpretation

An important consideration is that infantile colic probably has a multifactorial etiology [5, 12, 13], and it is therefore unlikely that one treatment would fit all, which can potentially reduce the overall mean effect size in a study like ours. Manipulative therapy is a treatment aimed at treating conditions in the musculoskeletal system. Hence, there might be subgroups of children with biomechanical problems who could potentially benefit more than others from this treatment [28]. We proposed this when planning the study [20], and investigations into potential treatment modifying factors will be the next step in the analyses of the study and will be reported in a separate article. This could potentially form the basis for a more stratified approach to the condition of infantile colic.

The reduction in duration of crying seen in the control group was substantial and probably reflects the natural cause of infantile colic. However, information and support for parents with crying babies is acknowledged as increasingly important [12], and in some guidelines is recommended as the cornerstone of intervention in families with a colicky child [5]. We found that, overall, more than 90% of parents were satisfied with their participation in the trial. This should also lead to consideration of the type of outcomes future studies should comprise, since other factors besides reduction in crying time may be important to the families [19, 24].

## Conclusion

In conclusion, we found that excessive crying was reduced by half an hour in favor of the group receiving chiropractic care compared with the control group, but not at a statistically significant level after adjustments. From a clinical perspective, the mean difference between the groups was small, but there were large individual differences, which emphasizes the need to investigate if subgroups of children, e.g. those with musculoskeletal problems, benefit more than others from chiropractic care.

## Data Availability

The datasets generated and/or analysed during the current study are not publicly available due to the policies of the Danish Data Protection Agency but are available from the corresponding author on reasonable request.

## Abbreviation

PI: Primary investigator
